# Improved Deformability of Co‐Free Medium Entropy Oxides by Controlled Entropy Destabilization

**DOI:** 10.1002/advs.78244

**Published:** 2026-10-15

**Authors:** Chang Liu, Ke Xu, Zhongxia Shang, Yang Chen, Debargha Paul, Yinghang Liu, Jeremy Gan, Shiyu Zhou, R. Edwin García, Xinghang Zhang, Haiyan Wang

**Affiliations:** ^1^ School of Materials Engineering Purdue University West Lafayette USA; ^2^ Birck Nanotechnology Center Purdue University West Lafayette USA; ^3^ School of Electrical and Computer Engineering Purdue University West Lafayette USA

**Keywords:** coherent Cu‐rich secondary phases, controlled entropy destabilization (CED), in situ micropillar compression, medium‐entropy oxide

## Abstract

High‐entropy oxides (HEOs), such as (CoCuMgNiZn)O, are attractive structural ceramics due to their compositional complexity and single‐phase stability. However, their intrinsic brittleness remains a major limitation. Here, we introduce a strategy termed as “controlled entropy destabilization” to activate defect‐mediated plasticity in a Co‐free medium‐entropy oxide (MEO) (CuMgNiZn)O. Spark plasma sintering produces a Rocksalt structure containing pre‐existing Cu‐rich secondary phases that act as defect sources during deformation. In situ SEM micropillar compression studies from room temperature to 600°C, combined with transmission electron microscopy and crystallographic orientation mapping, reveal a clear temperature‐dependent transition in deformation mechanisms. At room temperature, deformation is dominated by crack initiation with limited dislocation activity. At 400°C, thermally activated dislocation motion leads to delayed cracking and increased plastic strain accommodation. At 600°C, extensive dislocation glide and stacking‐fault formation produce pronounced plasticity and strain hardening. The fracture strain increases from 2.0% ± 0.3% at room temperature to 15.9% ± 0.1% at 600°C. These findings demonstrate that the controlled entropy destabilization approach can activate dislocation nucleation, migration, and storage, enabling intrinsic plasticity in multielement oxide ceramics at moderate temperatures.

## Graphical abstract

Controlled entropy destabilization activates defect‐mediated plasticity in Co‐free (CuMgNiZn)O by promoting pre‐existing Cu‐rich secondary phases that serve as defect sources. Temperature‐dependent dislocation activity evolves from limited motion at room temperature to extensive glide and stacking‐fault formation at 600 °C, enabling improved plasticity and strain hardening in multielement oxide ceramics.

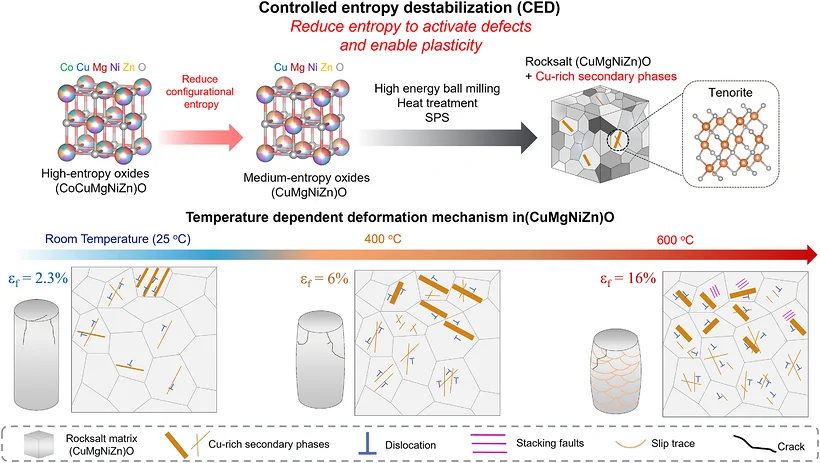

## 1 Introduction

Achieving plastic deformability in ceramics at room or moderate temperatures remains a long‐standing challenge because of their strong covalent bonding and the lack of effective mechanisms for strain accommodation [1]. Conventional toughening strategies such as grain refinement [2, 3], second‐phase reinforcement [4], phase transformation [5, 6], primarily rely on extrinsic crack shielding mechanisms rather than intrinsic plastic flow. Recent studies suggest that plasticity can be activated through defect engineering. Techniques such as spark plasma sintering (SPS) and flash sintering have been shown to introduce high densities of defects, including dislocations, stacking faults, and vacancies [7, 8, 9, 10], while preloading can further stimulate defect activity and dislocation motion [11]. These pre‐existing defects can enable plasticity in ceramics at moderate temperatures or even at room temperature.

High‐entropy oxides (HEOs) offer a different framework for tailoring plasticity in ceramics. By incorporating multiple cations on a single sublattice, configurational entropy lowers the Gibbs free energy of a homogeneous solid solution, giving rise to lattice distortion, sluggish diffusion, and solid‐solution strengthening, which have been associated with disrupt strain localization, delay crack propagation [12, 13, 14]. Recent studies have revealed that such compositional complexity can give rise to unexpected deformation behaviors. In Rocksalt (CoCuMgNiZn)O, room‐temperature plasticity has been directly observed in micron‐sized grain samples through dislocation‐mediated slip, with slip bands and activation of $\frac{1}{2}\langle 110\rangle \left\{1\bar{1}0\right\}$ slip systems [15]. Complementary micropillar compression studies further reveal that deformation is highly orientation dependent using Schmid factor analysis. Specifically, pillars along (001) and (101) activate dislocation slip, whereas (111) oriented pillars experience predominantly brittle fracture [16]. At elevated temperatures, the deformability becomes even more pronounced. Recent studies show that (CoCuMgNiZn)O undergoes superplastic deformation at 600°C–850°C with low creep stress exponents (*n* ≈ 0.2‐2.5), indicating grain boundary sliding dominated behavior. Shear‐thickening transitions occur at stresses above ∼9 MPa, associated with a shift toward solution‐precipitation mechanisms facilitated by Cu‐rich secondary phases [17]. Notably, superplastic forming can be achieved at relatively low pressures (∼70 kPa) and temperatures (∼800°C) [18]. More recently, in situ micropillar compression studies have provided direct evidence of temperature‐dependent plasticity in (CoCuMgNiZn)O, revealing the transition from brittle fracture to dislocation‐mediated plastic flow, accompanied by slip band formation, grain fragmentation, and the emergence of Cu‐rich phases that actively participate in strain accommodation [19]. However, excessive entropy driven stabilization could over homogenize the lattice and suppress local chemical and structural fluctuations, which could otherwise serve as alternative mechanisms for strain accommodation. This analysis raises an important question: could deliberately reducing configurational entropy to activate defect mediated mechanisms enable plastic deformation in ceramics?

Here we introduce a design strategy of “controlled entropy destabilization” for defect activation and plastic deformation in ceramics. Rather than maximizing entropy to enforce phase stability via dopant additions (e.g., Li, Na Fe) [20, 21, 22, 23, 24], we intentionally reduce the number of constituent elements while preserving the parent Rocksalt structure. This design is motivated by the distinct roles of individual cations in multicomponent Rocksalt oxides. Particularly, Co acts as a strong lattice stabilizer due to its intermediate ionic size, robust Co─O bonding, and favorable mixing thermodynamics that promote a uniform cation distribution and suppress local chemical segregation [25, 26]. Furthermore, Co is relatively costly and toxic for synthesis of oxides [27]. Exposure to cobalt powders and dust can cause respiratory issues, while the release of waste disposal may contaminate soil and water. By contrast, Cu exhibits a strong tendency for local enrichment and planar defect formation due to its pronounced Jahn‐Teller instability, which destabilizes ideal octahedral coordination and induces local lattice distortion [28]. Moreover, Cu valence fluctuations between Cu^2+^ and Cu^+^ enable local charge compensation and facilitating solute redistribution [29, 30]. By removing Co from the parent (CoCuMgNiZn)O high‐entropy oxide while retaining Cu, we intentionally reduce excessive lattice stabilization and activate Cu‐rich second phases.

Based on this design, we investigate the temperature‐dependent deformation behavior of (CuMgNiZn)O using in situ SEM micropillar compression combined with post‐mortem microscopy and precession electron diffraction (PED). By tuning configurational entropy, second phase could be used to promote dislocation nucleation, transmission, and work hardening, thereby opening a pathway toward more damage‐tolerant deformable ceramic materials. Although demonstrated in a specific oxide composition, this strategy can be applicable to other multicomponent oxide ceramics.

## 2 Experimental Methods

### 2.1 Processing and Synthesis

CuO (with 25–55 nm reported particle size, 99.95%), MgO (50 nm, 99.95%), NiO (18 nm, 99.98%), and ZnO (18 nm, 99.95%) nanosized powders were blended in equimolar amounts. The raw powders were mixed via high‐energy ball milling (HEBM) utilizing a SPEX 8000D mill for 30 hrs in an alumina jar with alumina media. The HEBM was done in 30 min intervals, interrupted by 10‐min pauses to avoid overheating and powder agglomerating. These powders were used to consolidate coarse‐grained samples using conventional sintering furnace at 900°C for 20 min. Then the powders were sintered by the spark plasma sintering (SPS) technique using SPS‐10, Thermal Technologies LLC. The powder was placed in a cylindrical graphite die with an inner diameter of 10 mm. A graphite foil was inserted into the inner wall to prevent the powder from adhering to the graphite tooling. The SPS system was powered on when the vacuum in the chamber reached 2 × 10^−2^ torr. The sintering temperature ramped to 800°C for 5 min at a heating rate of 200°C/min along with a pressure of 35 MPa. The relative density of the sintered specimen was measured to be 97% by the Archimedes method.

### 2.2 Microstructure Characterizations

The structure of SPS MEO was characterized by the x‐ray diffraction (XRD) technique by using a PANalytical Empyrean X'pert PRO MRD diffractometer with a 2 × Ge (220) hybrid monochromator to select Cu Kα1 in the 2θ—ω geometrical configuration. A Thermo Fisher Quanta 650 FEG scanning electron microscope and an FEI Talos 200X transmission electron microscope operated at 200 kV were utilized to analyze the microstructure of specimens. Grain sizes were estimated by the line intercept method over 200 grains based on transmission electron microscopy (TEM) images. TEM samples were manually prepared by grinding, polishing, dimpling and final polishing in an Ar ion milling system (PIPS ii, Gatan) at 2 kV. The crystallographic information embedded in the Inverse pole figure (IPF) and Kernel average misorientation (KAM) maps was generated by Precession electron diffraction (PED) equipment (ASTAR, NanoMEGAS, Belgium) installed in the FEI Talos 200X TEM microscope.

### 2.3 in situ Micropillar Compression Testing

The SPS MEO was manually polished on diamond paper (15 to 1 µm) and colloidal silica (20 nm) to minimize the surface roughness. The polished samples were mounted on a specimen stub to coat a thin layer of platinum at 40 mA for 60 s to enhance the electric conductivity in SEM. Micropillars with ∼3.5 µm in diameter and 7 µm in height (an aspect ratio of diameter to height of ∼ 1:2) were fabricated using a focused ion beam (FIB) in a Thermo Fisher Quanta 3D FEG scanning electron microscope. A series of decelerated currents (from 30 to 0.3 nA) was used to fabricate the pillars in the center of the initial SPS‐sintered sample, with a smooth surface and minimized tapering angle (average taper angle of 2°–3°). 5 pillars were tested at each temperature, including room temperature, 400°C, and 600°C. A Hysitron PI 88 × R PicoIndenter equipped with a piezoelectric actuator on the capacitive transducer was installed in Quanta 3D FEG microscope to perform in situ SEM micropillar compression tests. A 20‐µm diameter diamond flat punch was mounted on the transducer to collect force‐displacement data during compression. The specimen was tightly fixed by a V‐shape Mo clamp on a ceramic heating stage to maximize the heat transfer and minimize machine compliance. The temperature on both the heating stage and probe heater was ramped up simultaneously at a rate of 10°C/min and isothermally stabilized for 30 min to eliminate thermal drift. An average drift rate of less than 0.7 nm/s was monitored in the preloading process at each testing temperature. Each mechanical test was performed at a strain rate of 5 × 10^−3^ s^−1^ under displacement control mode. All compression tests were conducted under displacement‐controlled loading with a maximum displacement of 1400 nm, corresponding to approximately 20% strain. Tests were terminated upon catastrophic failure if failure occurred before reaching this displacement limit. Each stress‐strain curve shown in Figure 5 corresponds to an individual micropillar tested under identical conditions (same temperature, same strain rate, and comparable pillar geometry). For each temperature, 3–4 representative pillars are shown to illustrate the reproducibility and scatter inherent to small‐scale mechanical testing.

## 3 Results

Figure 1 summarizes the synthesis route and phase evolution of the (CuMgNiZn)O medium‐entropy oxide (MEO). The starting powders consist of multiple crystal structures including Rocksalt MgO and NiO, Tenorite CuO, and Wurtzite ZnO (Figure 1a). After HEBM for 30 hrs, the mixed oxide powders exhibit broadened diffraction peaks (Figure 1f), indicating severe lattice distortion and primary defect accumulation. After heat treatment at 900°C for 20 min, the Rocksalt phase remains dominant, while additional diffraction peaks indicate the presence of secondary phases. This phase separation is attributed to increased atomic mobility at high temperature and the reduced solid‐solution stability of the MEO system. The heat‐treated powders were subsequently consolidated by SPS. After SPS, the secondary‐phase peaks disappear and a single‐phase Rocksalt structure is obtained (Figure 1f), indicating the reincorporation of the secondary phases into the Rocksalt structure. The broader XRD peaks after SPS are attributed to increased lattice strain and defect accumulation, consistent with the TEM observations. Figure 1e illustrates the distribution of Mg, Ni, Cu, and Zn cations within the Rocksalt lattice. The corresponding crystallographic representation (Figure 1g) further confirms the Rocksalt structure for the 4‐element MEOs.

**FIGURE 1:**
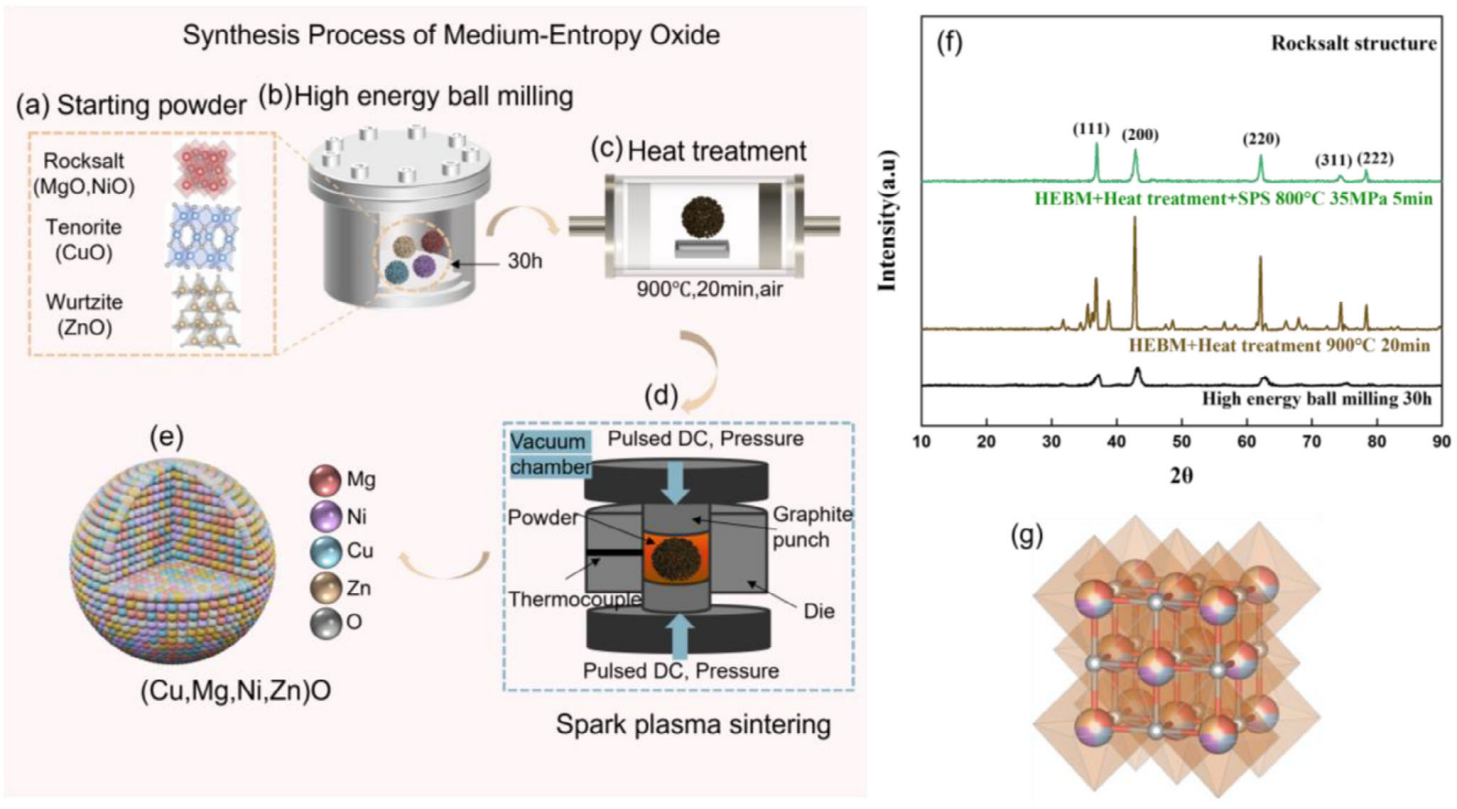
Synthesis process and phase evolution of the (CuMgNiZn)O medium‐entropy oxide (MEO). (a‐e) shows synthesis process of MEOs. (a) Crystal structures of the starting oxide powders. (b) High energy ball milling (HEBM) for 30 hrs to achieve thorough mixing and defect activation. (c) Heat treatment at 900°C for 20 min in air. (d) Spark plasma sintering (SPS) under pulsed DC and uniaxial pressure to consolidate the mixed powders. (e) Schematics showing the homogeneous distribution of Mg, Ni, Cu, and Zn cations in the rocksalt lattice. (f) X‐ray diffraction (XRD) patterns showing the structure evolution from the milled powders to the calcined mixture and finally to single‐phase rocksalt structure after SPS. (g) Rocksalt crystal structure of MEO.

Figure 2 presents the microstructure of (CuMgNiZn)O MEO synthesized by SPS. Bright‐field TEM image (Figure 2a) reveals a polycrystalline microstructure with equiaxed grains. The corresponding grain size distribution (Figure 2b) shows an average grain size of 1.40 ± 0.43 µm. Intragranular defects are frequently observed within individual grains as shown in Figure 2c,d. IPF map (Figure 2e) shows random grain orientation distribution without pronounced texture. KAM map (Figure 2f) shows prominent misorientation within the grain instead of along GBs, implying that strain is predominantly accommodated within the grains. GB map (Figure 2g) indicates that microstructure is dominated by high‐angle GBs. Geometrically necessary dislocation (GND) map (Figure 2h) shows localized high GNDs, consistent with KAM map (Figure 2f) and presence of intragranular defects in TEM image in Figure 2a. The SEM‐EDS elemental maps and EDS spectrum of the SPS‐sintered (CuMgNiZn)O sample are provided in Figure S1.

**FIGURE 2:**
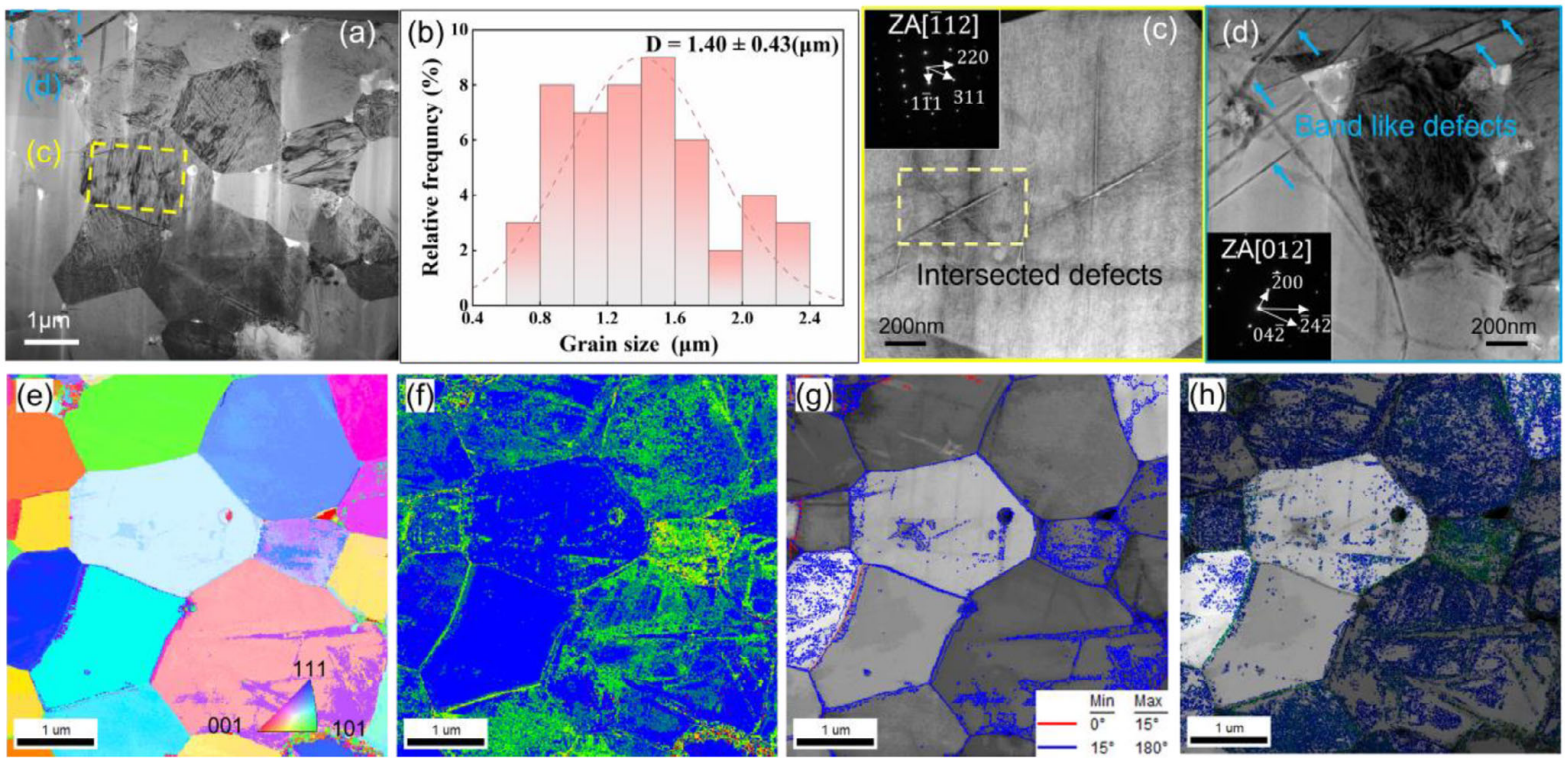
Microstructure of (CuMgNiZn)O MEO. (a) Bright‐field TEM image providing an overview of the specimen with polycrystalline microstructure. (b) Grain size distribution histogram indicating an average grain size of 1.40 ± 0.43 µm. (c) Scanning transmission electron microscopy (STEM) image showing intersected defects along [$\bar{1}12$] zone axis. (d) TEM image taken along the [012] zone axis showing band like planar defects (e) Inverse pole figure (IPF) map demonstrating a random grain orientation distribution without pronounced texture. (f) Kernel average misorientation (KAM) map showing localized misorientation associated with defect‐rich regions. (g) Grain Boundary (GB) map showing dominated high‐angle GBs. (h) Geometrically necessary dislocation (GND) density map indicating a localized high‐density of GNDs.

Figure 3 presents a detailed EDX analysis of planar defect regions exhibiting intersected (Figure 3a) and band‐like (Figure 3h) features. The intersected defects shown in Figure 3a have an estimated width of 13 nm, whereas the band‐like defect in Figure 3h exhibits a narrower width of 5 nm. A clear Cu‐rich feature is observed in both regions. Compared with the intersected defects (Figure 3c), the band‐like feature (Figure 3j) exhibits a prominently greater Cu concentration, ∼ 50 at% as shown by comparing the quantitative EDS line scan analysis in Figure 3g,n. The pronounced increase in Cu atomic fraction is accompanied by a simultaneous decrease in Zn, Mg and Ni contents.

**FIGURE 3:**
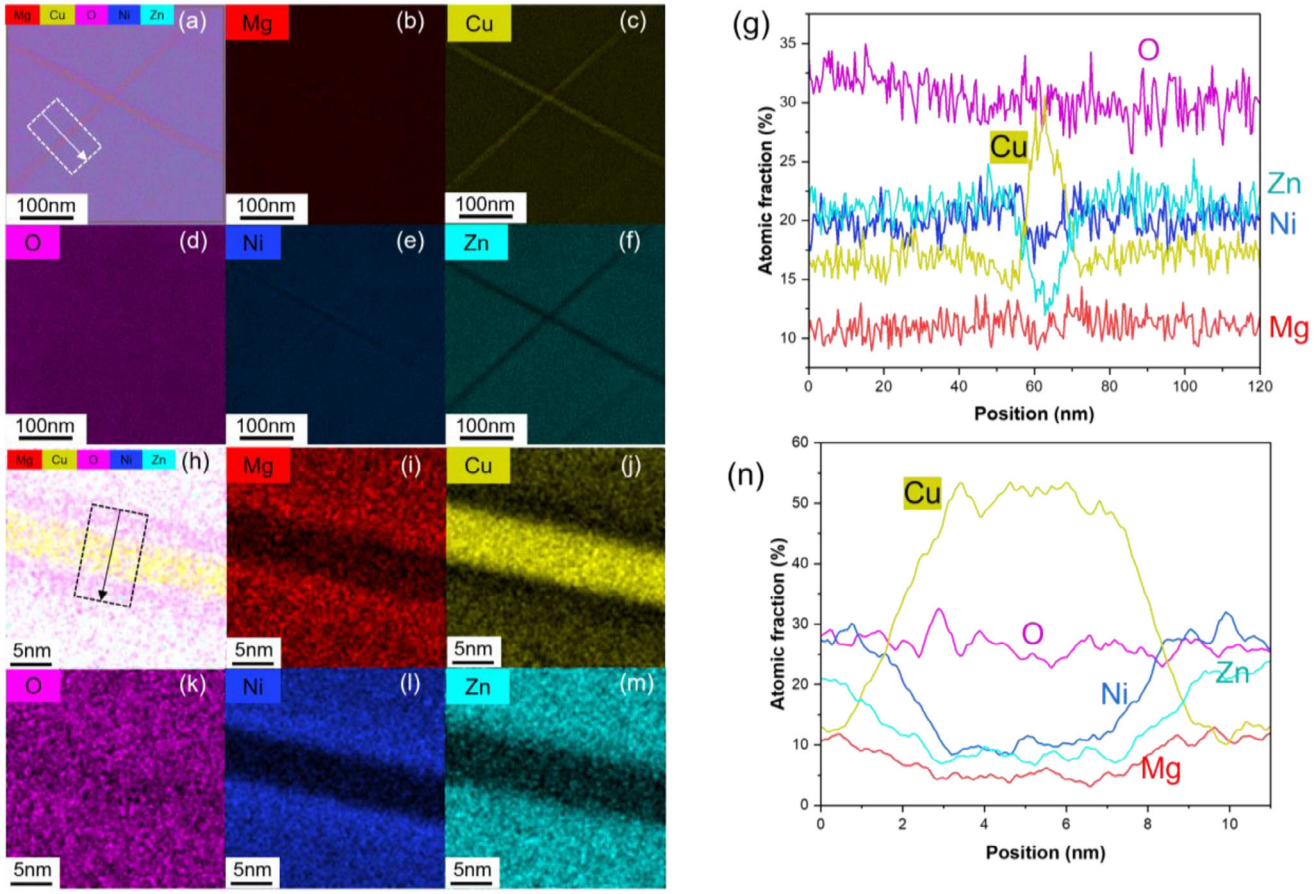
Energy dispersive spectroscopy (EDX) analysis of planar defect regions revealing Cu‐rich precipitation. (a–f) EDS overlay map and corresponding elemental maps acquired from an intersected defect region. (h–m) EDS overlay map and corresponding elemental maps obtained from a band‐like planar defect region. (g) and (n) EDX line‐scan profiles taken along the marked region in (a) and (h) respectively, showing pronounced Cu enrichment accompanied by local depletion of Zn and Ni.

Figure 4 shows atomic‐scale TEM and STEM characterization of Cu‐rich secondary phases before deformation. A cross‐over shaped Cu‐rich secondary phases in Figure 4a exhibits lattice modulation around 0.9 nm. Geometric phase analysis (GPA) strain mapping in Figure 4b reveals compressive shear strain (ε_xy_) localized within the cross‐over Cu‐rich secondary phases. An enlarged HRTEM image from the defect region in Figure 4c shows locally distorted lattice fringes with diffuse FFT streaking, consistent with lattice disorder and strain. Regions outside the X‐shaped defect in Figure 4d exhibit a highly‐ordered lattice with a 2H‐like ABAB stacking sequence, as confirmed by sharp FFT reflections. Cu‐rich band‐shaped secondary phases along the zone axis [001] in Figure 4e shows a coherent interface between precipitate and matrix with an angular relationship of approximately 84.9°. The corresponding GPA map in Figure 4f reveals a tensile strain band (ε_xx_) in the Cu‐rich secondary phases. Within the Cu‐rich secondary phases in Figure 4g, a lattice expansion with d(200) = 0.236 nm is measured, whereas regions outside Cu‐rich secondary phases in Figure 4h exhibit d(200) = 0.223 nm. Notably, there are no obvious pre‐existing dislocations. Based on these structural observations together with the compositional analysis, these nanoscale, compositionally distinct Cu‐rich regions that retain the Rocksalt structure are referred to as “Cu‐rich secondary phases” throughout this work, consistent with the terminology commonly used in high‐entropy/entropy‐stabilized oxide system. The different compositions and structures of these two Cu‐rich secondary phases may reflect different stages of Cu segregation. The cross‐over shaped region shows moderate Cu enrichment with pronounced lattice distortion, whereas the band‐shaped region exhibits stronger Cu enrichment and a more ordered coherent structure. Therefore, the cross‐over shaped region may represent a precursor state that evolves into the more Cu‐rich band‐shaped region through further Cu segregation and structural reorganization. However, this precursor relationship should be further confirmed using in situ TEM experiment.

**FIGURE 4:**
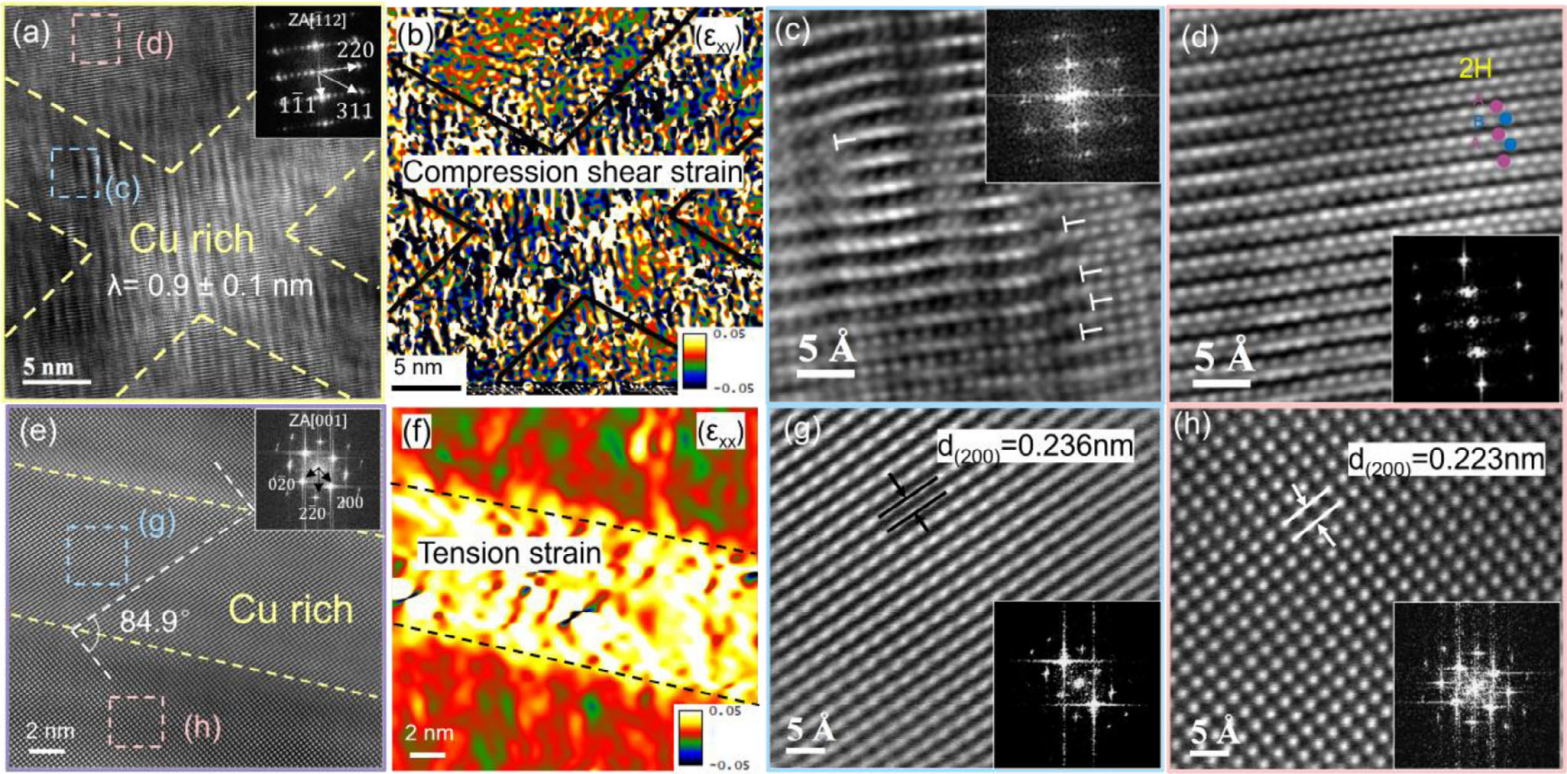
Atomic‐scale characterization of Cu‐rich defect regions before deformation. (a) High resolution TEM (HRTEM) image showing Cu‐rich (confirmed in Figure 3) secondary phases with lattice modulation around 0.9 nm estimated from FFT. The inset FFT taken along zone axis (ZA) $\bar{[1}12]$ indicates a distorted rocksalt structure. (b) Geometric phase analysis (GPA) strain map (ε_xy_) derived from Figure 4a demonstrating strong compressive shear strain in the defect region. (c) Enlarged HRTEM image from the box c in (a) revealing locally bent lattice fringes with inset FFT showing diffuse streaking. (d) HRTEM image taken outside the crossover secondary phases region in (a) exhibiting a 2H‐like ABAB stacking sequence. (e) High resolution STEM (HRSTEM) image showing a Cu‐rich secondary phases with a coherent interface from ZA [001]. (f) GPA strain map (ε_xx_) derived from Figure 4e revealing a tensile strain across the Cu‐rich secondary phases. (g) HRSTEM image from inside the secondary phases region showing d(200) is 0.236 nm, accompanied by sharp FFT reflections. (h) HRSTEM image outside the band‐defect region in (e) exhibits d(200) = 0.223 nm.

Figure 5 summarizes the mechanical behavior of SPS (CuMgNiZn)O tested by in situ micro‐compression at room temperature (RT), 400°C, and 600°C. The average ultimate compressive strength (UCS), yield stress, and fracture strain at RT, 400°C, and 600°C are summarized in Table S1 in the supporting information. At RT, the MEO (solid lines) shows a delayed stress drop compared with the HEO (dotted lines) in Figure 5a, indicating better deformability. Straight and long crack propagation and crack deflection on the pillar top in Figure 5b–d is observed. These pillars deformed by forming large cracks, with little plastic deformation. At 400°C, the plastic deformability of pillars has improved. The stress‐strain curves in Figure 5e exhibit noticeable non‐linear behavior accompanied by stress oscillations. When ε = 6%, SEM snapshots in Figure 5f–h reveal crack branching instead of large dominant cracks, suggesting repeated crack initiation, arrest, and branching during deformation. At 600°C, the plastic deformability of MEO pillars has increased substantially. Figure 5j was acquired after heating to 600°C and holding for thermal stabilization. During this heating/holding stage, some slip traces had already emerged, likely due to thermally activated relaxation of local residual stresses. The stress‐strain curves in Figure 5i display extended stress plateaus up to 16% strain. After compression, the slip traces became more abundant and evident (Figure 5k,l), indicating enhanced dislocation activity under mechanical loading, while cracking is largely suppressed.

**FIGURE 5:**
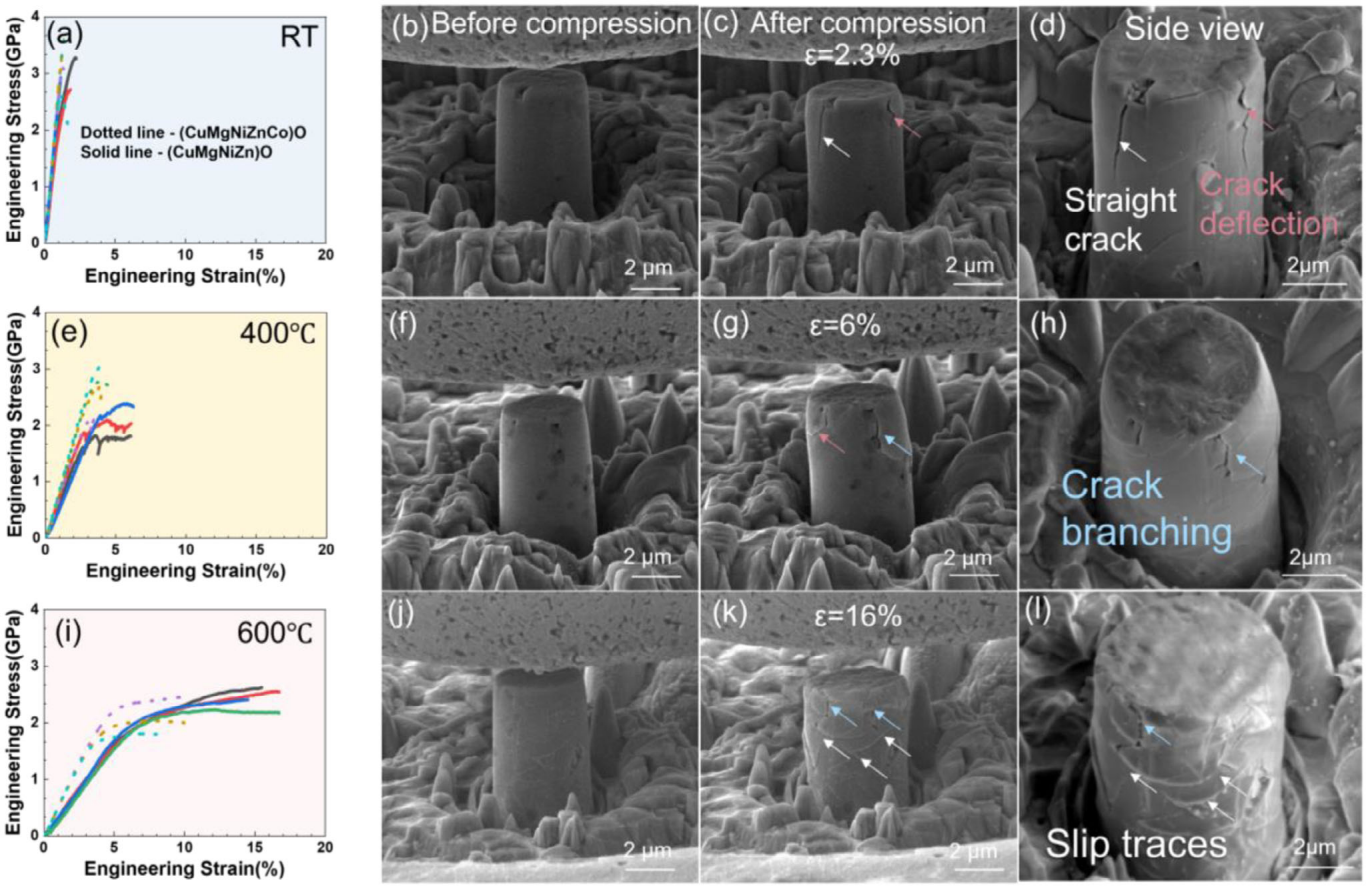
Mechanical behaviors of SPS MEO under in situ micro‐compression tests at room temperature (RT), 400°C, and 600°C at a constant strain rate of 5 × 10^−3^ S^−1^. (a,e,i) Engineering stress‐strain curves demonstrating the improved deformability of (CuMgNiZn)O (solid lines) compared with (CuMgNiZnCo)O (dotted lines). (b–d) SEM images acquired at RT (ε = 2.3%) showing straight crack propagation and crack deflection, indicating brittle fracture behavior. (f–h) SEM images of a pillar deformed at 400°C (ε = 6%) revealing crack branching, suggesting enhanced damage tolerance at elevated temperature. (j–l) SEM images acquired at 600°C (ε = 16%) showing extensive slip traces. The corresponding in situ SEM micropillar compression videos at RT, 400°C, and 600°C are provided as Movies S1, S2, and S3.

Post‐mortem TEM analyses were performed on the deformed micropillars after room‐temperature compression to a strain of 2.3% to elucidate the deformation‐induced microstructural evolution. Figure 6a presents a BF XTEM overview of the deformed pillar. An apparent dilation is observed near the pillar top region, while grain fragmentation and crack formation are predominantly found in the less‐deformed lower region. TEM micrograph from the highly deformed region (Figure 6b), acquired along the [$\bar{1}13$] zone axis, reveals the presence of band‐like Cu‐rich secondary phases extending over hundreds of nanometers. In comparison, the relatively undeformed region (Figure 6c), viewed along the zone axis [012], shows intersected Cu‐rich secondary phases. As shown in Figure 6d, the HRTEM image taken along the zone axis [$\bar{1}11$] from the deformed region reveals preserved lattice periodicity with locally distributed dislocations. The measured lattice spacing of the (202) planes is 0.22 nm, consistent with the Rocksalt structure. These observations show that deformation at RT is highly localized near the pillar top, where limited dislocation activity exists. Meanwhile, the interior region mainly accommodates deformation through grain fragmentation and crack formation.

**FIGURE 6:**
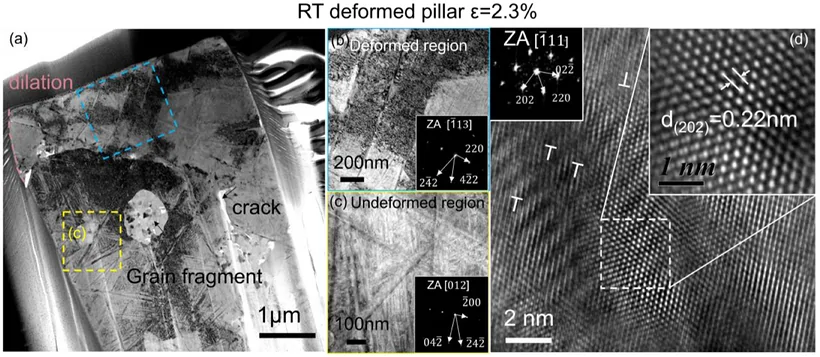
Post‐mortem TEM analyses of the (CuMgNiZn)O micropillar after RT compression to 2.3% of strain. (a) BF XTEM overview of the deformed pillar, showing apparent dilation near the top surface, while grain fragmentation and crack formation were observed in the less‐deformed region. (b) TEM image reveals band‐like Cu‐rich secondary phases and lots of strain accumulation along zone axis [$\bar{1}13$] in deformed region. (c) TEM image from a comparatively undeformed region along the zone axis [012], exhibiting intersected Cu‐rich secondary phases. (d) HRTEM image along the zone axis [$\bar{1}11$] in deformed region, showing preserved lattice periodicity with dislocations. The measured lattice spacing of the (202) planes is 0.22 nm.

Figure 7a shows a BF XTEM image taken from the top region of micropillar deformed to 6% strain at 400°C. Grain fragmentation and band‐like Cu‐rich secondary phases are observed near the pillar top without crack. Distinct cross‐over Cu‐rich secondary phases are observed in Figure 7b, characterized by an intersection angle of approximately 120°. Under the [111] zone axis, this angle is geometrically consistent with the projection of equivalent <110> directions on {111} planes. HRTEM image of the cross‐over Cu‐rich secondary phases in Figure 7c show strong lattice modulation with a periodicity of ∼0.9 nm estimated from the corresponding FFT, accompanied by pronounced local distortion and streaking features. An enlarged HRTEM image in Figure 7d reveals that the distorted region contains numerous dislocations. Figure 7e shows a BF‐TEM image acquired along the [$\bar{1}22$] zone axis, where extended band‐like Cu‐rich secondary phases are clearly visible across the grain. The corresponding DF‐TEM image in Figure 7f highlights a high density of dislocations surrounding and within these band‐like Cu‐rich secondary phases, indicating intensive dislocation activity associated with band formation. Furthermore, a high density of needle‐like Cu‐rich secondary phases are distributed within the dislocation‐rich zones in Figure 7g. An enlarged TEM image of this region reveals diffuse contrast and locally disordered microstructure.

**FIGURE 7:**
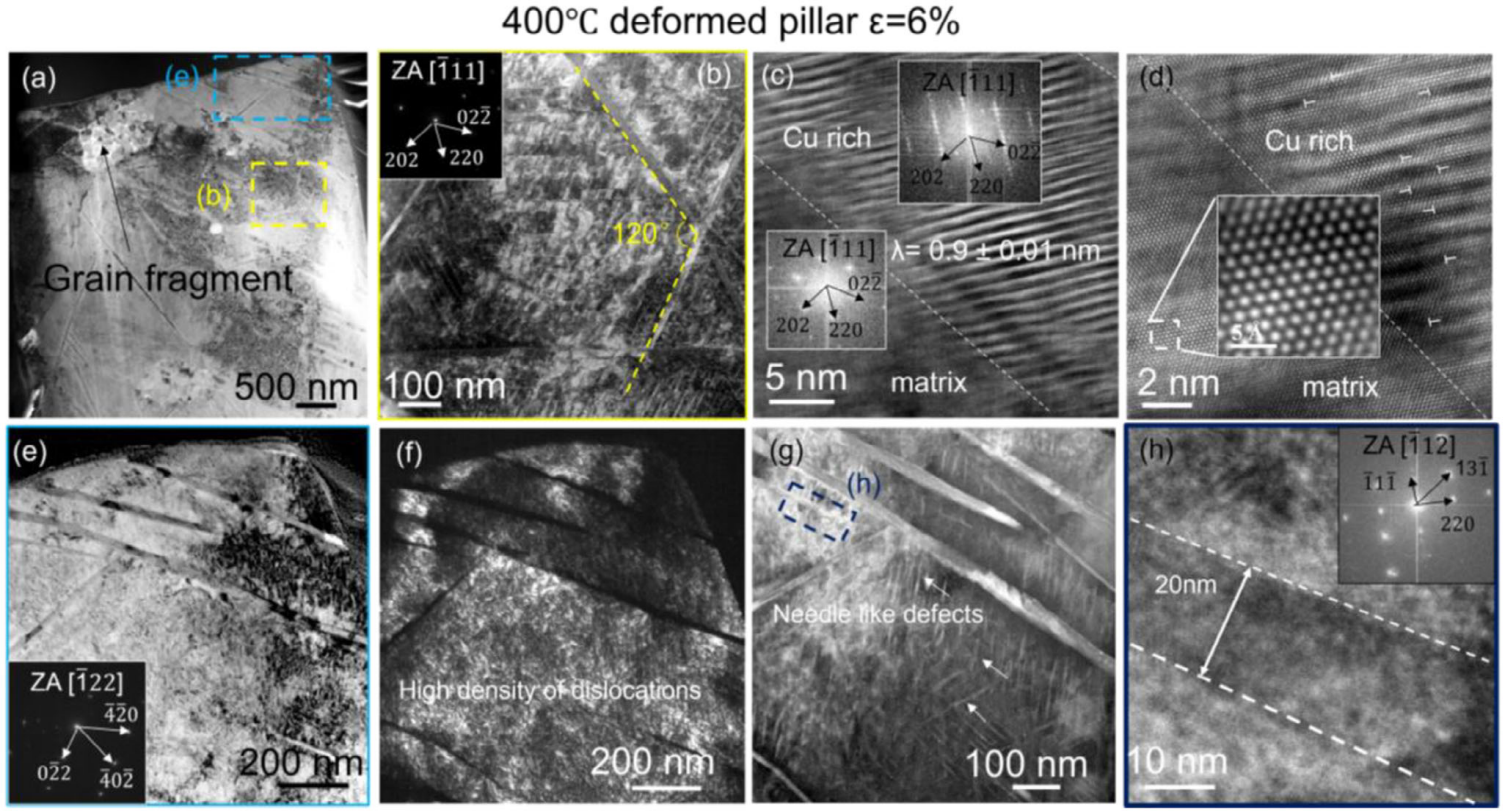
Post‐mortem TEM analyses of the micropillar after compression at 400°C to 6% strain. (a) BF XTEM image showing grain fragment and band‐like Cu‐rich secondary phases at the pillar top. (b) TEM image taken along the zone axis [$\bar{1}11$] revealing cross‐over Cu‐rich secondary phases with a characteristic angle of ∼120° in a less deformed region. (c) HRTEM image from [$\bar{1}11$] zone axis within the cross‐over defect in (b), showing pronounced lattice distortion with a lattice modulation of ∼0.9 nm estimated from the corresponding FFT inset. (d) HRTEM image of (c), where numerous dislocations are observed within the Cu‐rich secondary phases (e) BF TEM showing extended band‐like Cu‐rich secondary phases from zone axis [$\bar{1}22$]. (f) DF TEM image highlighting a high density of dislocations surrounding the Cu‐rich secondary phases. (g) STEM image revealing a high density of nanoscale needle‐like Cu‐rich secondary phases beneath the dislocation‐rich regions. (h) HRTEM image of the needle‐like Cu‐rich secondary phases, showing diffuse contrast and local structural disorder, with the corresponding FFT inset.

Figure 8 presents post‐mortem TEM analyses of the micropillar after compression to 16% strain at 600°C. The BF‐XTEM image in Figure 8a reveals multiple intersecting band‐like Cu‐rich secondary phases and localized grain fragmentation, indicating severe plastic deformation rather than brittle fracture. Notably, the grain fragmentation highlighted in the lower left region suggests intragranular rotation and grain refinement induced by high‐temperature deformation. BF‐TEM image in Figure 8b shows the band‐like Cu‐rich secondary phases consist of dense defect networks. Correspondingly, DF‐TEM image in Figure 8c reveals increased dislocation density concentrated around band‐like Cu‐rich secondary phases. In addition, needle‐like Cu‐rich secondary phases are frequently observed in Figure 8d. These needle‐shape Cu‐rich secondary phases exhibit a strong crystallographic orientation preference along <111> direction under the [011] zone axis. HRTEM image in Figure 8e further reveals the presence of stacking faults within the needle‐like Cu‐rich secondary phases, with an interplanar spacing of d_(111)_ = 0.21 nm, consistent with the FCC Rocksalt structure.

**FIGURE 8:**
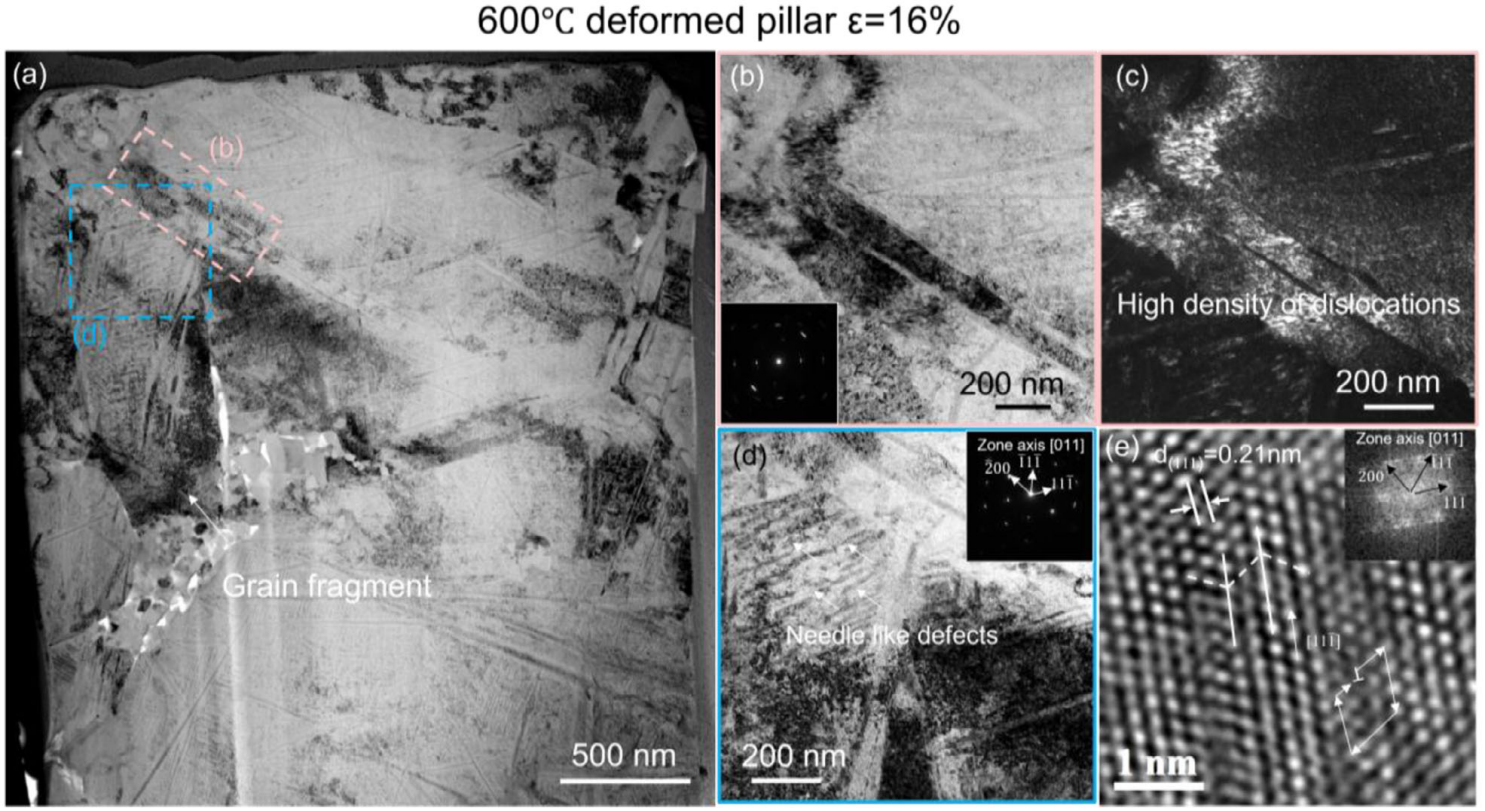
Post‐mortem TEM analyses of a micropillar after compression at 600°C to ε = 16%. (a) BF XTEM image showing planar Cu‐rich secondary phases and localized grain fragmentation. (b) BF TEM taken from box (a) revealing band‐shape Cu‐rich secondary phases with pronounced defect contrast. (c) DF TEM image showing high density of dislocations around the band‐shape Cu‐rich secondary phases. (d) BF TEM image acquired from box (a) highlighting needle‐like Cu‐rich secondary phases embedded within high density of dislocations under [011] zone axis. (e) HRTEM image revealing stacking faults along [$11\bar{1}$] in needle‐like Cu‐rich secondary phases region with d_(111)_ = 0.21 nm.

Figure 9 compares the ASTAR results obtained by Precession electron diffraction (PED) for the selected micropillars deformed at RT, 400, and 600°C. At RT, the IPF map (Figure 9a) reveals a complex grain orientation. Numerous low angle grain boundaries (LAGBs) (0‐15°) are observed within grains (Figure 9b). The KAM map (Figure 9c) shows corresponding misorientation near LAGBs. At 400°C, the IPF map (Figure 9e) exhibits significantly improved orientation continuity, with some grain refinement near the pillar top. The fraction of LAGBs decreased markedly to 6.2% (Figure 9f), while high angle grain boundaries dominated. The KAM map (Figure 9g) shows a more uniform distribution of misorientation. Some areas with high misorientation can be observed in the grain refinement region. At 600°C, the IPF map (Figure 9i) demonstrates enhanced grain orientation continuity, indicating more uniform microstructure. Similar to the pillar deformed at 400°C, the LAGB fraction in Figure 9j remains low (6.6%). The KAM map (Figure 9k) displays a relatively homogeneous distribution of misorientation. The GND density (as shown in Figure 9d,h,l) increases with test temperature and preferentially accumulates along grain boundaries and defect regions, suggesting enhanced dislocation multiplication and strain accommodation through defect‐mediated plasticity at elevated temperatures.

**FIGURE 9:**
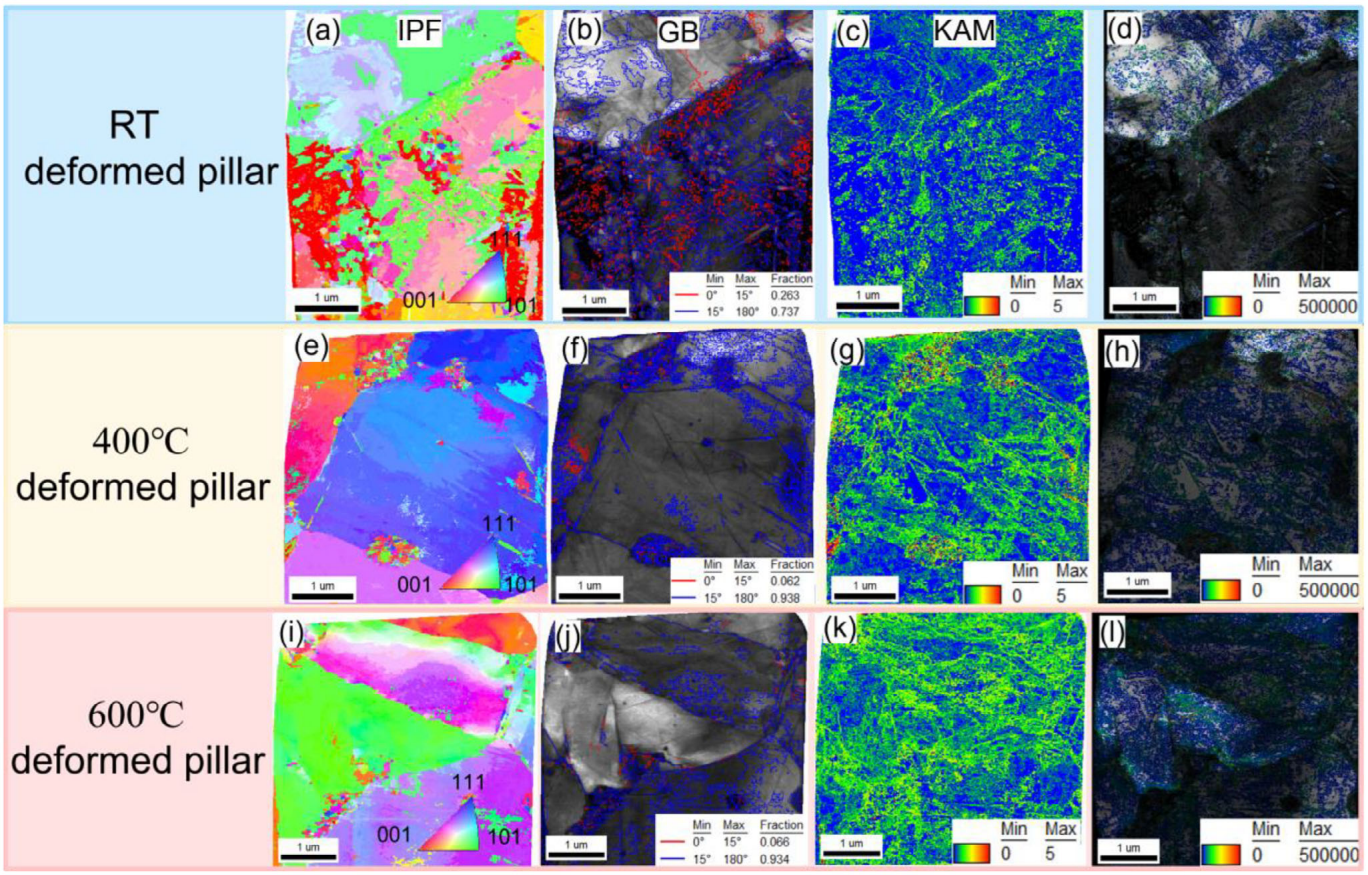
ASTAR analyses of the pillars deformed at RT, 400°C and 600°C. (a,e,i) IPF orientation maps. (b,f,j) Grain boundary (GB) maps overlaid on Image Quality (IQ) maps with boundary fractions. The red lines indicate low‐angle grain boundaries (LAGBs), and the blue lines indicate high‐angle grain boundaries (HAGBs). (c,g,k) KAM maps showing local lattice misorientation. (d,h,l) GND maps showing spatial variations in GND density. All maps are displayed at the same length scale.

## 4 Discussion

### 4.1 Single Phase Formation in Medium Entropy Oxide

Whether removal of Co destabilizes the single phase represents a central question of the present entropy‐destabilization strategy. XRD results (Figure 1f) show that SPS processed (CuMgNiZn)O maintains a single‐phase Rocksalt structure without detectable secondary phases. The stability of multicomponent oxide solid solutions is governed by the Gibbs free energy of mixing [31, 32]:

(1) $$\Delta \;G_{mix}=\Delta H_{mix}-T\Delta S_{config}$$

where Δ*H_mix_* denotes the enthalpy of mixing, T denotes the absolute temperature, and configuration entropy is expressed as [31, 32]:

(2) $$\Delta \;S_{config}=-R\sum_{i}x_{i}lnx_{i}$$

where *x_i_* denotes the atomic fraction of each element and *R* denotes the gas constant. For equimolar compositions, reducing the oxide system from five to four cations decreases configurational entropy from *Rln*5 to *Rln*4. Previous studies have shown that removal of individual cations from the prototypical Rocksalt high‐entropy oxide can still yield single‐phase four‐component solid solutions [33]. Moreover, stable Rocksalt oxides have been reported at entropy levels as low as ∼0.95R [34], indicating that high entropy is not a strict prerequisite for phase stabilization.

Importantly, phase stability in Rocksalt multicomponent oxides dependents not solely on entropy magnitude but also on the specific roles and structural compatibility of constituent cations. Recent studies indicate that the stability of Rocksalt structure is mainly controlled by cations favoring octahedral coordination, such as Mg^2^^+^ and Ni^2^^+^, which provide a stable structural framework. Zn^2^^+^ can adopt Rocksalt coordination under multicomponent mixing conditions, contributing to solid‐solution formation. However, Co is not essential for maintaining the Rocksalt structure but mainly assists chemical homogenization during synthesis [33]. Therefore, although removal of Co may slightly reduce lattice stability, the remaining cation network remains sufficient to form a homogeneous Rocksalt phase.

### 4.2 Nature of the Cu‐Rich Secondary Phases Regions

Although XRD confirms formation of a single‐phase Rocksalt structure after SPS, TEM reveals Cu‐rich secondary phases embedded within the matrix (Figure 2, 3, 4). Similar Cu‐rich secondary phases have been widely reported in Rocksalt entropy‐stabilized (CoCuMgNiZn)O [35, 36, 37, 38, 39]. However, a quantitative description that links the Cu‐rich secondary phases stress field to solute redistribution has remained largely unexplored. Here we address this gap using the classical Cottrell‐Eshelby elastic interaction framework.

KAM and GND analyses (Figure 2f‐h) reveal pronounced misorientation and localized strain gradient associated with cross‐over and band‐like Cu‐rich secondary phases. GPA strain mapping (Figure 4) further reveals tensile and compressive strain fields surrounding these planar Cu‐rich secondary phases, indicating prominent localized stress within the lattice. The elastic interaction energy between a solute and a Cu‐rich secondary phases stress field can be expressed as [40, 41, 42]:

(3) $$\Delta E=-\sigma_{ij}\varepsilon_{ij}^{*}V$$

where σ_*ij*_ represents the local stress associated with Cu‐rich secondary phases, $\varepsilon_{ij}^{*}$ denotes the eigenstrain introduced by solute substitution, and *V* is the effective interaction volume associated with the substitutional solute atom. Segregation becomes energetically favorable when the product $\sigma_{ij}\varepsilon_{ij}^{*}$ is positive (Δ*E* < 0), resulting in a reduction of elastic strain energy. Consequently, Cu‐rich secondary phases act as sinks for solutes to accommodate lattice strain. Among the constituent cations, Cu^2^^+^ produces the largest lattice distortion due to its Jahn–Teller active octahedral coordination [33, 43, 44], leading to a larger eigenstrain $\left(\varepsilon_{ij}^{*}\right)$ compared with Mg^2^^+^, Ni^2^^+^, and Zn^2^^+^. Consequently, segregation of Cu to planar Cu‐rich secondary phases most effectively reduces local elastic energy. In contrast, Mg^2+^ exhibits a nearly symmetric ionic configuration and moderate ionic size mismatch, generating limited lattice distortion and thus a weak elastic driving force for segregation [45]. As Cu preferentially occupies Cu‐rich secondary phases, Ni‐ and Zn‐rich species are correspondingly redistributed into the surrounding matrix to maintain local structural and compositional equilibrium. In addition to the thermodynamic driving force, kinetic factors may further promote segregation. Cu^2^^+^ is known to exhibit relatively greater cation mobility than Mg^2+^, Ni^2+^, Zn^2+^ in Rocksalt oxides due to its Jahn‐Teller induced lattice distortion and weaker bonding environment [46]. The relatively higher diffusivity enables rapid redistribution of Cu toward defect strain fields within the short SPS sintering duration.

HRTEM images (Figure 4a–d) show that the Cu‐rich secondary phases exhibit pronounced lattice distortion accompanied by embedded dislocations and locally modified 2H‐type ABAB stacking sequences. For the Rocksalt structure ($d_{111}=\frac{a}{\sqrt{3}}\;$), $d_{111}^{matrix}=0.250nm$, $d_{111}^{Cu-rich}=0.265nm$, corresponding to *a_matrix_* =  0.433*nm* and *a*_*cu* − *rich*_ =  0.459*nm* and yielding a local misfit following the classical definition of Frank and van der Merwe [47, 48, 49, 50]:

(4) $$f=\frac{a_{cu-rich}-\;a_{matrix}}{a_{matrix}}\;\approx 6\%$$

Such a large misfit exceeds the coherent elastic limit. The associated coherency strain energy of a constrained Cu‐rich secondary phases can be approximated as [51, 52, 53, 54, 55]:

(5) $$\gamma_{coh}∼\frac{E}{2\left(1-\nu \right)}f^{2}h$$

where *h* is the Cu‐rich secondary phases thickness, *E* is the Young's modulus, ν is the Poisson's ratio. Using *h* ≈ 8.4*nm* (measured from Figure 4a), *E* ≈ 180*GPa*(calculated from nanoindentation), and ν  =  0.25 [56, 57, 58], we obtain γ_*coh*_ ≈ 3.6 *Jm* ^−2^. This value is larger than typical grain‐boundary energies in ceramics, which are commonly in the range of 0.5 − 1.3*Jm* ^−2^ [59, 60], even comparable with Alumina (2.5 ± 0.73*Jm* ^−2^) [60]. The system therefore lowers its free energy through defect‐assisted relaxation, consistent with the dislocations and stacking modulations observed in Figure 4a–d.

With increasing Cu enrichment, the initially distorted region evolves into a continuous Cu‐rich secondary phases (Figure 4e,f). GPA strain mapping shows that the strain is greater inside the band core (ε_x__x_ ≈ 0.04) and gradually decreases toward the band boundary (ε_x__x_ ≈ ∼0.01), indicating strain redistribution. The absence of periodic misfit dislocations across the band/matrix boundary indicates that the interface remains largely coherent. Such a coherent strain region can redistribute local deformation, reduce interfacial stress concentration, and delay strain localization, thereby promoting more stable plastic flow compared with shear‐dominated cross‐over shaped defects. However, few dislocations are observed in Figure 4e–h, which indicates that most of the lattice mismatch is accommodated internally within the Cu‐rich band prior to interface stabilization.

### 4.3 Deformation Mechanisms

#### 4.3.1 Brittle Feature at RT

Despite the presence of Cu‐rich secondary phases and pre‐existing defects that may act as potential dislocation nucleation sites, the overall plastic strain at RT remains low. TEM observations reveal microcracks with sizes (a) of approximately 350 nm. The critical condition for crack propagation can be estimated using linear elastic fracture mechanics [61, 62]:

(6) $$K_{I}=\sigma \sqrt{\pi a}$$

where *K_I_* is the stress intensity factor at the crack tip, σ is the applied stress, *a* is the characteristic crack size. Crack propagation occurs when *K_I_* reaches the fracture toughness *K_IC_*. Using the estimated indentation‐derived fracture toughness $K_{IC}\approx 1.9\;\mathrm{MPa}\sqrt{m}$, ​we estimate that σ_*c*_ ≈ 1.8*GPa*. This critical stress is lower than the stress at which the first surface crack is observed during in‐situ SEM compression (∼2.7 GPa), suggesting that crack initiation likely occurred within the pillar interior before being detected at the surface. Consequently, deformation is governed by internal crack nucleation and subsequent crack propagation rather than sustained dislocation plasticity.

#### 4.3.2 Deformation Transition Regime at 400°C

Figure 10 summarizes the temperature dependence of the yield stress measured in the micropillar compression tests. The yield stress was determined from the stress‐strain curves as the point where the loading curve first deviates from the elastic behavior [63]. The average yield stress (Table S1) decreases from 1.8 GPa at RT to 1.4 GPa at 400°C, followed by a moderate increase to 1.6 GPa at 600°C. The moderate recovery in yield stress at 600°C may be associated with the increased contribution of Cu‐rich secondary phases strengthening. At elevated temperature, enhanced dislocation activity promotes interactions between mobile dislocations and the Cu‐rich secondary phase regions, which can act as obstacles to dislocation motion. Together with the grain refinement observed in Figure 9i, these defect‐dislocation interactions may partially offset thermal softening. For thermally activated plastic flow, the shear yield stress can be described by [63, 64]:

(7) $$\tau_{y}=\tau_{p}\;-\frac{kT}{V}ln\left(\frac{\dot{\gamma_{0}}}{\dot{\gamma }}\right)$$

where τ_*p*_ represents the Peierls stress corresponding to the lattice resistance at 0 K, *V* is the activation volume, *k* is the Boltzmann constant, $\dot{\gamma }$ is the shear strain rate and $\dot{\gamma_{0}}$ is a reference strain rate associated with dislocation mobility. Converting the normal stress to shear stress using $\tau_{y}=\frac{\sigma_{y}}{\sqrt{3}}\;$ gives a shear stress sensitivity of $m=\frac{d\tau_{y}}{dT}\;\approx -0.62MPaK^{-1}$, differentiation yields $m=-\frac{k}{V}\ln\left(\frac{\dot{\gamma_{0}}}{\dot{\gamma }}\right)$. Using the experimental strain rate $\dot{\varepsilon }=5\times 10^{-3}s^{-1}$, shear strain rate $\dot{\gamma }=\sqrt{3}\;\dot{\varepsilon }=8.66\times 10^{-3}s^{-1}$, and a typical reference strain rate $\dot{\gamma_{0}}∼10^{6}-10^{7}s^{-1}$ [65, 66], the activation volume can be estimated as *V* ≈ (4.1 − 4.7) × 10^−28^ *m* ^3^. For a Rocksalt lattice with *a* ≈ 0.42*nm* (calculated based on XRD) and Burgers vector $b=a/\sqrt{2}\approx 0.3nm$, this corresponds to *V* ≈ 15 − 18*b* ^3^. Activation volumes on the order of 10–100b^3^ are typically associated with dislocation motion interacting with short‐range obstacles such as solute atoms [63, 64, 65, 67, 68, 69, 70, 71]. Based on the thermally activated flow relation [40, 70, 72]:

(8) $$\dot{\gamma }=\dot{\gamma_{0}}\;exp\left(-\frac{\Delta G}{kT}\right)$$

**FIGURE 10:**
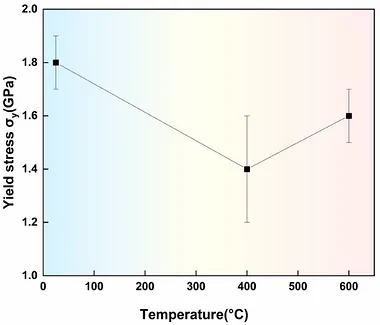
Temperature dependence of yield stress of the medium‐entropy oxide during micropillar compression.

The apparent activation energy for dislocation motion can be estimated as $\Delta G\approx kTln(\frac{\dot{\gamma_{0}}}{\dot{\gamma }})$. Using *T*  =  673 K (400°C) gives Δ*G* ≈ (1.1‐1.2) eV, which is characteristic of dislocation motion overcoming local lattice obstacles in oxides. This interpretation is consistent with the TEM observations at 400°C, which reveal dense defect structures, including high density of dislocations (Figure 7d,f), Cu‐rich secondary phases (Figure 7c,d), and needle‐like Cu‐rich secondary phases (Figure 7g), which generate strong local stress fields and provide short‐range barriers for dislocation motion.

#### 4.3.3 High‐Temperature Plastic Regime at 600°C

At 600°C, the stress‐strain curves exhibit pronounced strain hardening accompanied by multiple slip traces (Figure 5i,l). TEM observations further reveal the formation of stacking faults (Figure 8e). These observations indicate that plastic deformation at this temperature is dominated by extensive dislocation activity and defect interactions. The strain hardening behavior can be described using the classical Taylor dislocation hardening relation [73]:

(9) $$\sigma =\sigma_{0}+\alpha MGb\sqrt{\rho }$$

where σ_0_ represents the lattice friction stress, which can be approximated by the yield stress at the onset of plastic deformation, α is the dislocation interaction coefficient (typically 0.3 [74]), *M* is the Taylor factor for polycrystals (*M* ≈ 3 [74]), *G* is the shear modulus (*G* ≈ 88.6*GPa* for Rocksalt oxides [75, 76]), *b* is the Burgers vector (*b* ≈ 0.3 *nm*), and ρ is the dislocation density. Using the experimentally measured stress increase from approximately 1.6 GPa at yielding to ∼2.4 GPa at ∼16% strain, the dislocation density is estimated to be ρ ≈ 1.1 × 10^15^ *m* ^−2^. This value indicates substantial dislocation multiplication and storage during deformation at 600°C. The formation of stacking faults observed in HRTEM further supports this interpretation. In Rocksalt oxides, dislocation glide on {111} planes can locally produce stacking faults through partial dislocation activity. As deformation proceeds, interactions between mobile dislocations, stacking faults, and nanoscale Cu‐rich secondary phases create strong obstacles to dislocation motion. These interactions promote dislocation accumulation and enhance strain hardening.

Overall, the temperature dependent deformation mechanisms are schematically summarized in Figure 11, highlighting the transition from crack‐dominated deformation at RT to dislocation assisted plasticity at 400°C and stacking fault/dislocation network mediated plasticity at 600°C.

**FIGURE 11:**
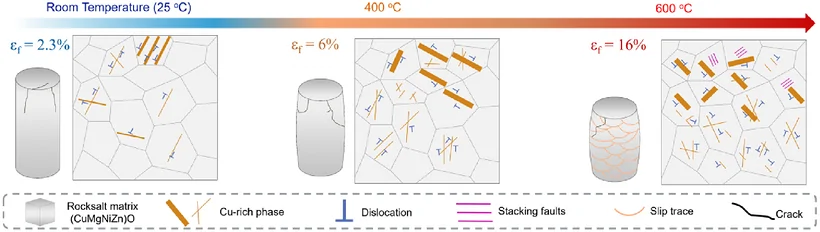
Schematic illustration of temperature dependent deformation mechanisms in (CuMgNiZn)O MEO. At room temperature (25°C), deformation is dominated by crack initiation with limited dislocation activity, resulting in a fracture strain of 2.3%. At 400°C, thermally activated dislocation motion and Cu‐rich phase‐assisted defect interactions delay crack propagation and increase the fracture strain to 6%. At 600°C, extensive dislocation glide, stacking‐fault formation, and distributed slip traces enable pronounced plasticity with a fracture strain of 16%.

### 4.4 Comparison With (CoCuMgNiZn)O and a Classical Rocksalt Structured MgO

It is noted that the micropillar compression tests for (CuMgNiZn)O and (CoCuMgNiZn)O were conducted under the same experimental conditions, including identical FIB fabrication procedures, pillar dimensions, strain rate, and heating stage. Therefore, the mechanical responses of these two material systems can be directly compared.

A direct comparison with the five‐component (CoCuMgNiZn)O system in our previous work [19] reveals that removing Co enhances plastic deformability without compromising the mechanical strength. At room temperature, the fracture strain increases from 1.5% ± 0.3% to 2.0% ± 0.3% (∼1.3 ×) while maintaining comparable strength (∼3.2 to 3.0 GPa). The advantage becomes more pronounced at elevated temperatures. At 400°C, strain to failure increases from 4.5% ± 0.5% to 6.1% ± 0.1% (∼1.4×) with a moderate strength reduction (2.7 to 2.1 GPa), and at 600°C, failure strain increases from 9.9% ± 0.9% to beyond 15.9% ± 0.1% (∼1.6×) with enhanced flow strength (2.1 to 2.4 GPa). Correspondingly, the stress‐strain plots for the four‐component system exhibit pronounced serrated flow at 400°C (Figure 5e) and stronger work hardening at 600°C (Figure 5i), indicating improved defect‐mediated plasticity. These results demonstrate that reducing configurational complexity can promote strain accommodation, making MEOs promising candidates for achieving enhanced plasticity (∼60% increase in strain) without sacrificing strength.

Using the Vickers indentation method established in our previous work [19], the HEO exhibited a hardness of 7.44 ± 0.27 GPa and a fracture toughness of 1.42 ± 0.02 MPa·$\sqrt{m}$. In comparison, the MEO exhibited a higher hardness of 8.05 ± 0.21 GPa and a substantially improved fracture toughness of 1.92 ± 0.06 MPa·$\sqrt{m}$, corresponding to increases of approximately 8.2% and 35.2%, respectively. As shown in Figure S2, cracks in the HEO propagates along relatively straight paths, whereas crack deflection is observed in the MEO.

MgO, a classical Rocksalt structured oxide, provides a useful reference for evaluating mechanical behavior of the MEO in this work. The deformation response of MgO is strongly dependent on crystal orientation, microstructure, temperature, and specimen size. For example, [100]‐oriented MgO single crystals can undergo approximately 6% plastic deformation at room temperature before fracture [77], whereas bulk polycrystalline MgO exhibits only approximately 0.6% total strain at fracture at room temperature and approximately 2% above 600°C [78]. Increasing temperature promotes dislocation mobility and activates additional slip systems, leading to improved plastic deformation [79, 80]. In comparison, the present MEO exhibits enhanced deformability, reaching ∼15.9% strain at 600°C with strain hardening. Unlike single phase MgO, the MEO contains Cu‐rich secondary phases that can act as both dislocation sources and barriers, facilitating dislocation activity and accumulation during deformation.

## 5 Conclusion

In this work, enhanced plastic deformability in the Co‐free medium‐entropy oxide (CuMgNiZn)O is achieved through controlled entropy destabilization. The formation of nanoscale Cu‐rich secondary phases introduces local lattice distortion and internal stress fields that strongly influence the deformation behavior. With increasing temperature, the deformation mechanism evolves from the crack‐dominated brittle fracture at room temperature to the thermally activated dislocation glide at 400°C, and further to stacking‐fault mediated plasticity and dislocation network induced hardening at 600°C. These defect interactions promote dislocation nucleation, transport, and storage, enabling significant strain hardening and large plastic strain at elevated temperatures. The results demonstrate that entropy destabilization can activate defect‐assisted plasticity in multicomponent oxides, providing an effective strategy to overcome the intrinsic brittleness of oxide ceramics.

## Author Contributions

**Chang Liu**: Conceptualization, Methodology, Validation, Investigation, Data curation, Formal analysis, Writing – original draft. **Ke Xu**: Methodology, Investigation, Data curation, Writing – review & editing. **Zhongxia Shang**: Investigation, Data curation, Writing – review & editing. **Yang Chen**: Investigation, Data curation, Writing – review & editing. **Debargha Paul**: Investigation, Writing – review & editing. **Yinghang Liu**: Investigation, Writing – review & editing. **Jeremy Gan**: Investigation, Writing – review & editing. **Shiyu Zhou**: Investigation, Writing – review & editing. **R. Edwin García**: Supervision, Project administration. **Xinghang Zhang**: Supervision, Funding acquisition, Writing – review & editing. **Haiyan Wang**: Conceptualization, Supervision, Funding acquisition, Writing – review & editing.

## Conflicts of Interest

The authors declare no conflicts of interest.

## Supporting information




**Supporting File 1**: advs78244‐sup‐0001‐SuppMat.docx.

**Supporting File 2**: advs78244‐sup‐0002‐MovieS1.mp4.

**Supporting File 3**: advs78244‐sup‐0003‐MovieS2.mp4.

**Supporting File 4**: advs78244‐sup‐0004‐MovieS3.mp4.

## Data Availability

The data that support the findings of this study are available on request from the corresponding author. The data are not publicly available due to privacy or ethical restrictions.
