# Supplementary material for: Improved Deformability of Co‐Free Medium Entropy Oxides by Controlled Entropy Destabilization
Source: Adv Sci (Weinh). 2026 Oct 15:e78244. Online ahead of print. doi: 10.1002/advs.78244 (PMC13673067; doi:10.1002/advs.78244)
Supplement: Supplementary file 1 [file ADVS-9999-e78244-s004.docx]

***Improved deformability of Co-free medium entropy oxides by controlled entropy destabilization***

Chang Liu^1^, Ke Xu^1^, Zhongxia Shang^2^, Yang Chen^1^, Debargha Paul^1^, Yinghang Liu^1^, Jeremy Gan^1^, Shiyu Zhou^1^, R. Edwin García^1^, Xinghang Zhang^1,^ and Haiyan Wang^1,3, *^

^1^School of Materials Engineering, Purdue University, West Lafayette, IN 47907, USA.

^2^Birck Nanotechnology Center, Purdue University, West Lafayette, IN 47906, USA.

^3^School of Electrical and Computer Engineering, Purdue University, West Lafayette, IN 47907, USA.

*Corresponding author: [hwang00@purdue.edu](mailto:hwang00@purdue.edu)

**Supplementary Materials Include:**

Table S1. Summary of average UCS, yield stress, σy (GPa), fracture strain at different temperatures.

Figure S1. SEM-EDS analysis of the medium-entropy oxide after SPS.

Figure S2. Comparison of crack propagation behavior in HEO and MEO ceramics.

Movie S1. *In situ* SEM micropillar compression test and load vs. displacement curve for (CuMgNiZn)O at RT

Movie S2. *In situ* SEM micropillar compression test and load vs. displacement curve for (CuMgNiZn)O at 400 ^o^C

Movie S3. *In situ* SEM micropillar compression test and load vs. displacement curve for (CuMgNiZn) O at 600 ^o^C

**Table S1. Summary of average UCS, yield stress, σ_y_ (GPa), fracture strain at different temperatures.**

|  | RT | 400°C | 600°C |
| --- | --- | --- | --- |
| Average $USC$ (GPa) | 3 ± 0.4 | 2.1 ± 0.3 | 2.4 ± 0.2 |
| Yield stress, $\sigma_{y}$ (GPa) | 1.8 ± 0.1 | 1.4 ± 0.2 | 1.6 ± 0.1 |
| Fracture strain, $\varepsilon_{f}$ (%) | 2 ± 0.3 | 6.1 ± 0.1 | 15.9 ± 0.1 |

Figure S1. SEM-EDS analysis of the medium-entropy oxide after SPS. (a-e) Elemental maps of Cu, Mg, Ni, O, and Zn, respectively. (f) Corresponding combined map, and (g) EDS spectrum acquired from the mapped area. The elemental maps show a relatively uniform distribution of the constituent elements. The measured composition is 12.7 at.% Cu, 12.1 at.% Mg, 12.1 at.% Ni, 13.1 at.% Zn, and 50.0 at.% O.

Figure S2. Comparison of crack propagation behavior in HEO and MEO ceramics. (a) Representative crack morphology in the HEO showing relatively straight crack propagation. (b) Representative crack morphology in the MEO exhibiting crack deflection indicated by the black arrows. The inset provides a magnified view of a crack-deflection region.
